# NTRK-rearranged spindle cell neoplasm of the female genital tract: case report and literature review

**DOI:** 10.3389/fonc.2025.1525722

**Published:** 2025-08-27

**Authors:** Lulu Feng, Lei Li, Yanmei He, Wei Jiang

**Affiliations:** ^1^ Department of Pathology, West China Second University Hospital, Sichuan University, Chengdu, China; ^2^ Key Laboratory of Birth Defects and Related Diseases of Women and Children, Sichuan University, Ministry of Education, Chengdu, China

**Keywords:** cervix, tumor, NTRK, NGS, prognosis

## Abstract

NTRK-rearranged spindle cell neoplasm is a rare subtype of soft tissue sarcoma that occasionally arises in the lower female genital tract. Accurate diagnosis is clinically important, as these tumors generally display low-grade malignant behavior and may respond to TRK inhibitor therapy. We report a rare case of cervical NTRK-rearranged spindle cell neoplasm in a 40-year-old woman who presented with abnormal vaginal bleeding. An initial biopsy of the cervical mass suggested a diagnosis of sarcoma. The patient subsequently underwent a total hysterectomy with bilateral salpingo-oophorectomy. Histological examination revealed spindle-shaped tumor cells diffusely infiltrating the cervical stroma in a solid growth pattern. Immunohistochemistry demonstrated diffuse expression of TRK and CD34. Fluorescence *in situ* hybridization (FISH) detected rearrangement of the *NTRK1* gene, and RNA-based next-generation sequencing confirmed a *TPM3::NTRK1* fusion gene. To date, only 61 cases of NTRK-rearranged spindle cell neoplasm in the female genital tract have been reported. Here, we present a new case with a detailed description of the clinical presentation, histopathological and immunophenotypic characteristics, molecular findings, and clinical outcome. Comparative analysis with previously reported cases suggests a possible correlation between *NTRK* fusion type and patient prognosis. Specifically, tumors with *NTRK1* fusions tend to present at earlier stages and are associated with more favorable outcomes. These findings highlight the potential value of tailoring clinical management strategies based on fusion type.

## Introduction

Uterine sarcomas represent a heterogeneous group of malignant mesenchymal tumors, with endometrial stromal sarcoma and leiomyosarcoma being the most common subtypes. Advances in molecular pathology have led to the identification of several novel sarcoma entities, including NTRK-rearranged spindle cell neoplasms (1), which can also occur in the female genital tract, particularly in the cervix (2–6). It is characterized by a “fibrosarcoma-like” spindle cell morphology and recurrent rearrangements involving the NTRK gene family. The histological features often overlap with those of other soft tissue tumors, posing significant diagnostic challenges, particularly in the absence of molecular genetic testing. Although recent studies have broadened the morphological and immunophenotypic spectrum of these tumors, prognostically relevant features remain undefined, and their biological behavior is still not well understood (7). Accurate identification holds clinical significance, as patients with recurrent or metastatic disease may benefit from targeted therapy using TRK inhibitors (8, 9). In this report, we describe a rare case of cervical NTRK-rearranged spindle cell neoplasm with detailed clinicopathological, immunophenotypic, and molecular features, aiming to improve diagnostic recognition and provide insights into the biological and therapeutic implications of this tumor type.

## Case presentation

A 40-year-old woman was admitted to the hospital with abnormal vaginal bleeding. Ultrasound imaging revealed an irregular, weakly echogenic mass in the cervix, measuring approximately 7.9 cm×6.7 cm×8.0 cm, with indistinct margins and prominent internal vascularity. Surgical excision of the cervical tumor was performed via a transvaginal approach. Gross examination revealed a mass predominantly located in the anterior lip of the cervix. Histopathological evaluation suggested a diagnosis of sarcoma. The patient subsequently underwent total hysterectomy with bilateral salpingo-oophorectomy, pelvic lymphadenectomy, and para-aortic lymph node sampling.

## Materials and methods

Histopathological examination was independently conducted by two gynecological pathologists. Details of the primary antibodies used for immunohistochemical (IHC) staining are provided in **Supplementary Table 1**. Immunostaining procedures were carried out according to standardized laboratory protocols and antibody manufacturer instructions, including the routine use of appropriate positive and negative controls.

Fluorescence *in situ* hybridization (FISH) analysis was carried out using a commercial dual-color break-apart probe kit targeting the *NTRK1*, *NTRK2*, and *NTRK3* genes (Guangzhou LBP Medicine Science & Technology Co., Ltd., Guangzhou, China). Formalin-fixed paraffin-embedded tissue sections were deparaffinized and digested with pepsin at 37°C for 9 minutes. Co-denaturation of tissue sections and probes was performed at 85°C for 5 minutes, followed by overnight hybridization at 37°C. After stringent post-hybridization washes, nuclei were counterstained with 4′,6-diamidino-2-phenylindole (DAPI), and slides were mounted with coverslips. A tumor cell was considered positive for gene rearrangement if distinct red and green fluorescent signals (break-apart pattern) were observed, indicating disruption of the *NTRK* gene locus. At least 100 tumor cells were evaluated per case, and a specimen was considered positive when >10% of cells exhibited break-apart signals.

RNA-based next-generation sequencing (RNA-NGS) was conducted by GenePlus Technology (Beijing, China). Total RNA was extracted using the RNeasy FFPE Kit (Qiagen, Cat. No. 73504) according to the manufacturer’s protocol. RNA integrity and concentration were assessed with the Agilent 2100 Bioanalyzer (Agilent Technologies, WA, USA). Sequencing libraries were constructed and subjected to 150 bp paired-end sequencing using the DNBSEQ-T7 platform (GenePlus, Beijing, China). Raw sequencing data were filtered to remove low-quality reads and adapter sequences. High-quality reads were subsequently aligned to the human reference genome (hg19) using the STAR aligner. Fusions were detected by a customized version of Arriba 1.1.0 and annotated by in house software annoFilterArriba (version:1.0.0) with NCBI release 104 database. A gene fusion event was confirmed when ≥5 high-quality, unique reads spanned the fusion breakpoint, and ≥3 of those reads had unique start sites. All final candidate fusions were manually verified with the integrative genomics viewer browser. A series of quality control metrics was computed by using RNA-SeQC assessment. A threshold of ≥ 80 million mapped reads and ≥ 10 million junction reads per sample was set. The genome was visualized using IGV software (GeneVis v1.2.3) (10, 11).

A literature review was conducted to identify previously published cases of NTRK-rearranged uterine sarcoma. English-language articles were retrieved from the PubMed database using the keywords “NTRK” combined with “uterus”, “uterine”, “cervix”, “cervical”, or “female genital tract”.

Statistical analysis of the literature-derived data was performed using SPSS software (version 25.0). Data are presented as mean ± standard deviation ( $\bar{\text{x}}\pm \text{s}$ ). For comparisons of means between two groups, the independent-sample t-test was used if assumptions of normality and homogeneity of variance were met. Otherwise, the nonparametric Wilcoxon rank-sum test was applied. A two-sided *p*-value < 0.05 was considered statistically significant.

## Results

**Figure 1:**
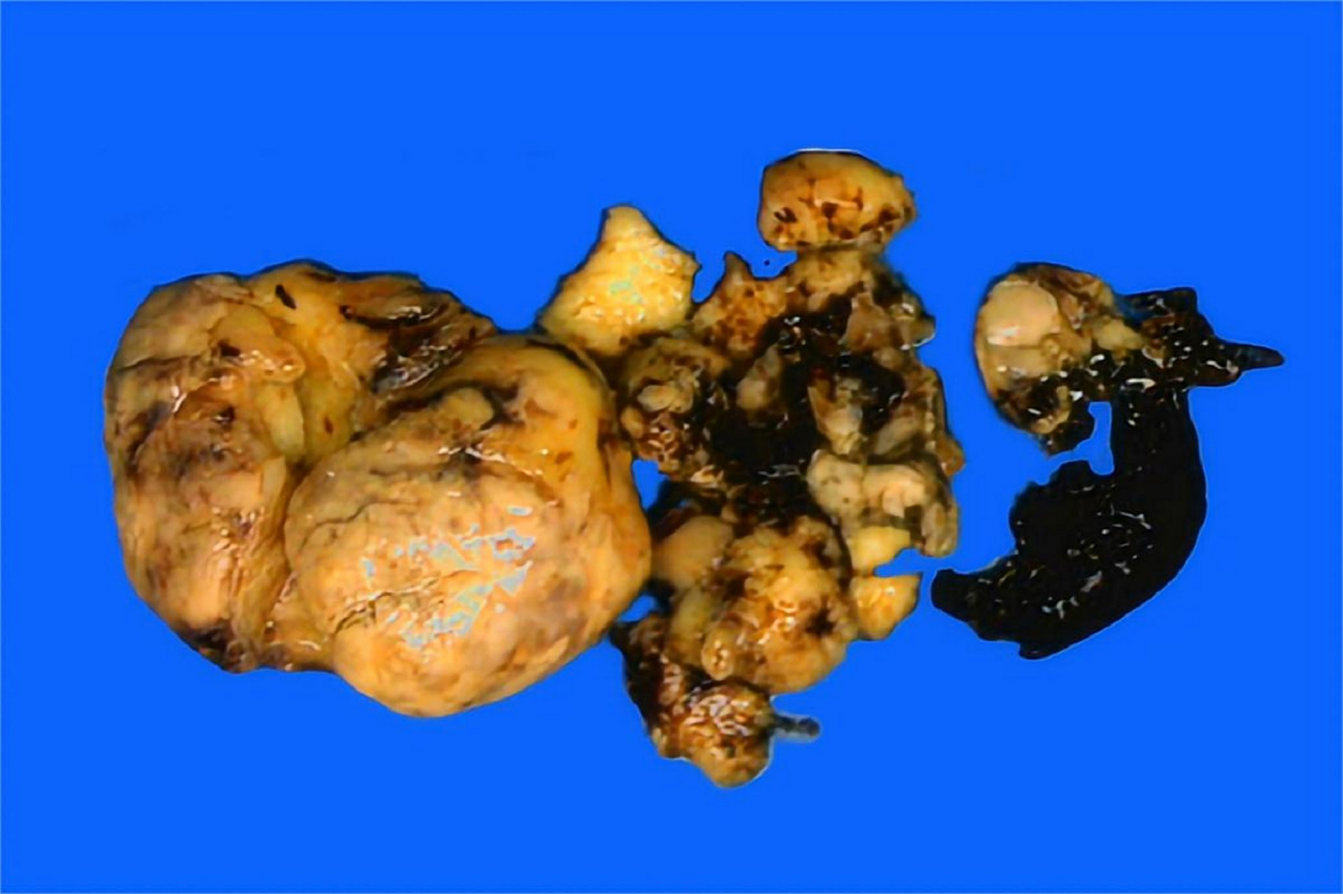
Grossly, the solid tumor presented a greyish-yellow cut surface with focal necrosis and hemorrhage.

**Figure 2:**
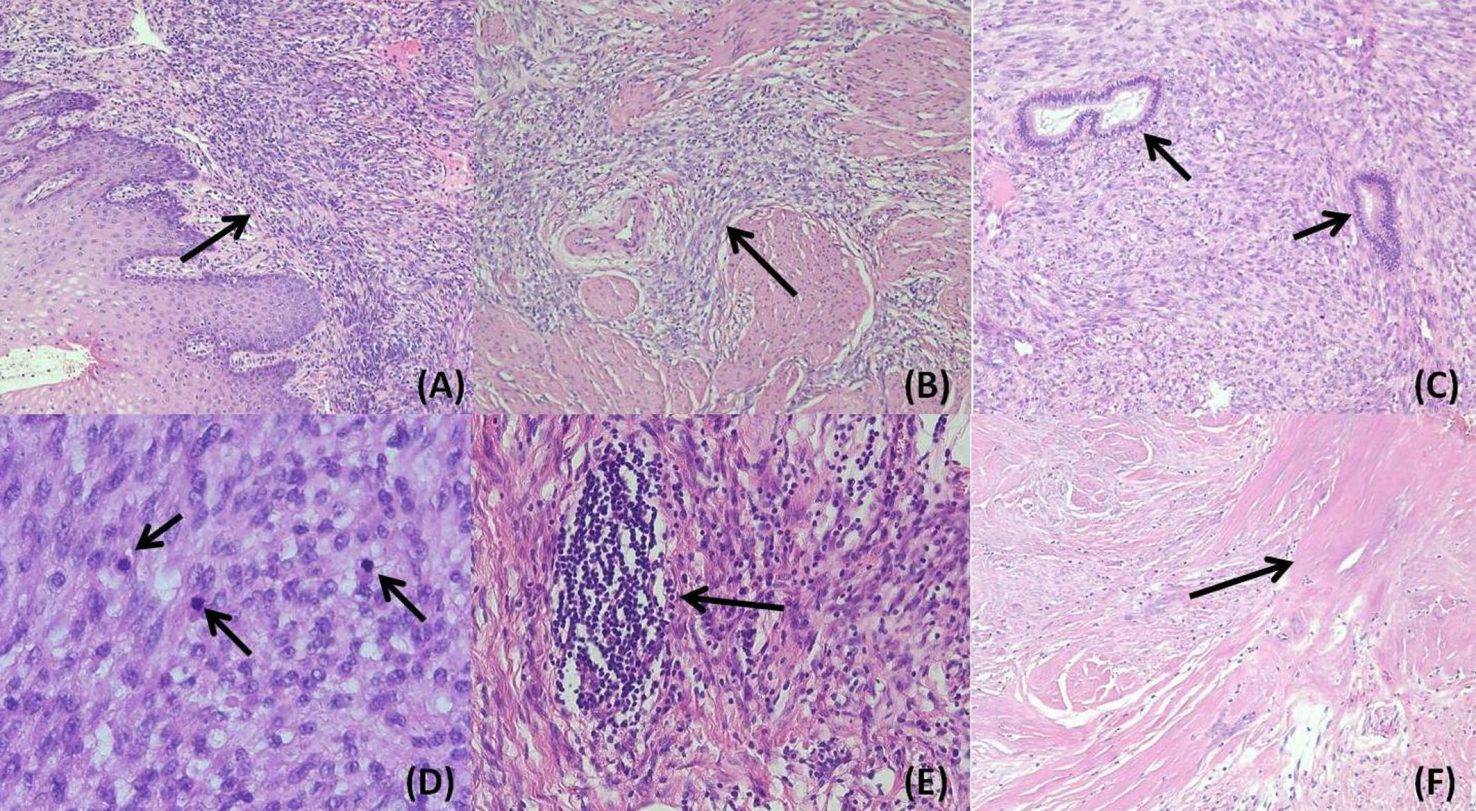
In the hematoxylin&eosin slide, the tumor exhibited a sheet-like growth pattern underneath the cervical mucosa **(A)** 100×magnification) and infiltrated the cervical stroma **(B)** magnification:100×). Residual endocervical glands were found among the tumor components **(C)** magnification:100×). The tumor consisted of spindle or oval cells with mild atypia and abundant mitoses **(D)** magnification:400×). Lymphocyte aggregation **(E)** magnification:200×) and necrosis **(F)** magnification:100×) could be found focally.

Gross examination revealed a solid, greyish-yellow tumor measuring 9.5 cm×7.3 cm×5.5 cm, and the tumor infiltrated approximately 50% of the cervical stroma (**Figure 1**). Microscopically, the tumor exhibited a diffuse, solid growth pattern with scattered residual endocervical glands. The tumor cells were relatively uniform, displaying spindle- or oval-shaped nuclei with mild nuclear atypia. Mitotic activity was brisk, with up to 25 mitoses per 10 high-power fields (HPFs). The tumor demonstrated variable-sized intratumoral blood vessels and focal lymphocytic infiltration (**Figure 2**). A total of 25 lymph nodes were dissected, including 12 from the left pelvic region, 12 from the right pelvic region, and 1 from the para-aortic region. No evidence of metastasis was identified.

**Figure 3:**
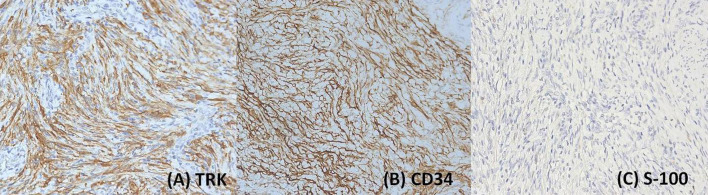
Tumor cells exhibited diffuse and strong immunostaining for TRK **(A)** magnification:200×) and CD34 **(B)** magnification:200×), while showing negative staining for S-100 (magnification: **(C)** 200×).

Immunohistochemical analysis showed diffuse and strong expression of TRK and CD34 in tumor cells. Focal and weak positivity was observed for cyclin D1, desmin, smooth muscle actin (SMA), CD10, estrogen receptor (ER), and progesterone receptor (PR). Staining for S-100, caldesmon, calponin, MyoD1, myogenin, BCOR, and ALK was negative (**Figure 3**). P53 staining showed a wild-type pattern, and the Ki67 proliferation index was approximately 15%. Based on the immunophenotype, uterine leiomyosarcoma, endometrial stromal sarcoma (ESS), rhabdomyosarcoma, and inflammatory myofibroblastic tumor (IMT) were excluded.

**Figure 4:**
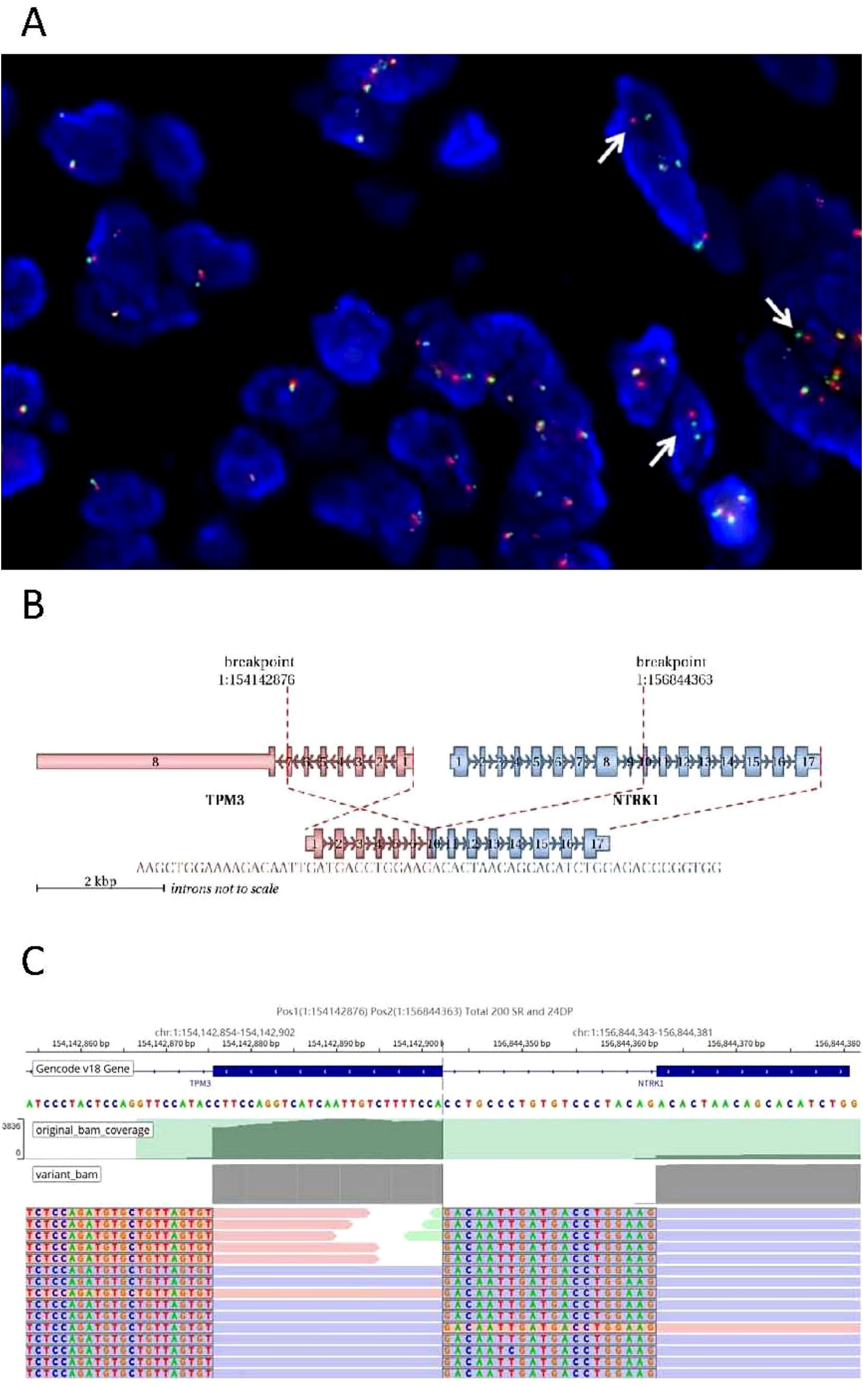
TPM3-NTRK1 fusion analysis. **(A)** Breakage of the NTRK1 gene was detected by FISH, which showed separate red and green signals in some tumor cells (arrow). **(B)** Schematic diagram of detailed fusion site of TPM3 and NTRK1. **(C)** NGS results showed a breakpoint of fusion. original bam coverge (Raw depth, including both mutated and non-mutated alleles); variant bam (Raw depth, including only mutated alleles); SR (Split Read): This refers to a sequencing read whose segments are mapped to different genomic locations. Such mapping indicates that the read spans a potential breakpoint, which may be caused by structural variations such as deletions, insertions, inversions, or translocations, resulting in discontinuity in the genomic sequence; DP (Discordant Pair): This refers to a pair of paired-end sequencing reads whose mapping does not conform to the expected insert size or orientation (e.g., the reads are oriented in opposite directions, or are mapped at an abnormal distance apart). A typical instance is when each read of a pair maps to two different genes, strongly suggesting the presence of a structural variant such as a translocation between these genes. SR and DP represent the most commonly used and critical types of bioinformatic evidence for the detection of genomic structural variations (SVs).

The FISH analysis demonstrated separated red and green fluorescence signals for the NTRK1 probe in tumor cells, indicating a rearrangement of the NTRK1 gene (**Figure 4A**). Targeted RNA sequencing was used to assess rearrangements involving a panel of 555 genes associated with tumorigenesis and tumor progression.A TPM3::NTRK1 gene fusion (NM_001043353.1/NM_002529.3) was detected in the tumor sample, with the specific fusion breakpoint located at exon 7 of TPM3 and exon 10 of NTRK1 (**Figure 4B**). A total of 224 supporting reads were identified (**Figure 4C**). This fusion is considered actionable for targeted therapy.

According to the 2018 International Federation of Gynecology and Obstetrics (FIGO) staging system, the tumor was classified as stage IB3 (pT1b3N0M0). The patient received adjuvant chemotherapy consisting of ifosfamide (1 g/m²/day, days 1-3), epirubicin (20 mg/m²/day, days 1-3), and cisplatin (20 mg/m²/day, days 1-3), all administered via intravenous infusion. The patient is currently undergoing adjuvant radiotherapy. No targeted therapy has been administered.

### Literature review and survival analysis

A total of 20 publications encompassing 61 cases of NTRK-rearranged spindle cell neoplasms in the female genital tract were included in the analysis. Among these, 54 cases were located in the uterine cervix and 7 in the uterine corpus. The clinicopathological features of all 61 patients are summarized in **Supplementary Table 2**. Data from the literature were statistically analyzed using SPSS software version 25.0, with a p-value of <0.05 considered statistically significant.

The mean age at diagnosis was 39 years (range: 13–69 years), and the average tumor size was 7.0 cm (range: 1.3–23.0 cm). At the time of diagnosis, most tumors were confined to the uterus, with 88% of patients classified as stage IA or IB according to the FIGO system. The average mitotic count was 12 per 10 HPFs, ranging from 0 to 50. Tumor necrosis was present in 40% of cases, and lymphovascular invasion was identified in 20%. Follow-up data were available for 50 patients. Among them, 26 patients (52%) had no evidence of disease (NED), while 7 patients (14%) died of disease (disease-specific death, DSD). In stage IA patients, 83% (10/12) were NED, and 17% (2/12) were alive with disease (AWD); notably, two patients in this group developed distant metastases involving the pleura and bone. Among patients at stage IB, 57% (12/21) were NED, 19% (4/21) were AWD, and 24% (5/21) died of disease. Only five patients were diagnosed at more advanced stages, including stage IIA (n = 2), stage IIB (n = 2), and stage IVB (n = 1) (**Table 1**).

**Table 1:** Summary of the clinical, morphologic, and molecular findings in our own case and the literature cohort (n=61).

Variable	Literature cohort	Our Case
Clinical		
Age (years)	Mean: 39 (range: 13-69)	40
Anatomic location	Cervix[56/61 (92%))]/corpus[5/61 (8%)]	cervix
Size (cm)	Mean: 7 (range: 1.3-23.0)	8
FIGO stage	IA[13/42 (31%)]/IB[24/42 (57%)]/IIA[2/42 (5%)]/IIB[2/42 (5%)]/IVB[1/42 (2%)]	IB3
Morphology		
Atypia	Mild-moderate[45/58 (78%)]/Severe[13/58 (22%)]	Mild-moderate
Mitoses	Mean: 12/10HPFs (range: 0-50/10HPFs)	25
Tumor necrosis	20/50 (40%)	Present
Lymphovascular invasion	7/44 (16%)	Absent
Molecular findings		
*NTRK1* (75%)	*TPM3*[24/41 (59%)]/*TPR*[10/41 (24%)]/*C16orf72*[2/41 (5%)]/*IR-F2BP2*[2/41 (5%)]/*LMNA*, *NUMA1*, *TRIM67*	*TPM3::NTRK1*
*NTRK2* (1%)	*WWOX*	\
*NRTK3* (24%)	*SPECC1L*[7/13 (54%)], *EML4*[3/13 (23%)], *TFG*, *STR-N*, *RBPMS*	\
Outcome		
Recurrence	18/50,36%	NO
outcome	NED[32/50 (64%)]/AWD[11/50 (22%)]/DSD[7/50 (14%)]	NED

AWD, indicates alive with disease; DSD, disease-specific death; NED, no evidence of disease; FIGO, International Federationof Gynecology and Obstetrics.

Molecular detection revealed that *NTRK1* fusions were the most common type (44/59, 75%), followed by *NTRK3* fusions (14/59, 24%) and a single *NTRK2* fusion (1%). Various fusion types were identified (**Table 1**), among which *TPM3::NTRK1* was the most common type (24/41, 59%). The comparative analysis demonstrated that tumors harboring *NTRK3* fusions were significantly larger than those with *NTRK1* fusions (mean diameter: 10.4 cm vs. 5.5 cm, *p* = 0.011) and more often presented at advanced FIGO stages (>IB vs. ≤IB, *p* = 0.016). Furthermore, chi-square analysis revealed a significantly higher disease-specific mortality rate in patients with *NTRK3* fusions compared to those with *NTRK1* fusions (33% vs. 6%, *p* = 0.028), suggesting a poorer prognosis associated with *NTRK3* fusion tumors (**Table 2**). Although *NTRK3* fusion tumors also exhibited a higher mitotic index and increased recurrence rates, these differences did not reach statistical significance. No significant differences were observed between the *NTRK1* and *NTRK3* fusion groups in terms of patient age, presence of lymphovascular invasion, or tumor necrosis.

**Table 2:** Comparison of clinicopathologic features between *NTRK1*-rearranged group(n=44) and *NTRK3*-rearranged group (n=14)^#^.

Variable	*NTRK1* (n=44)	*NTRK3*(n=14)	p
Mean age (years) ( $\bar{\text{x}}\pm \text{s}$ )	37.1 ± 10.8	36.2 ± 13.2	0.826
Stage			0.016^*^
≤IB	27/29 (93%)	7/12 (58%)	
>IB	2/29 (7%)	5/12 (42%)	
Mean size (cm) ( $\bar{\text{x}}\pm \text{s}$ )	5.5 ± 3.3	10.4 ± 5.7	0.011^*^
Mean mitotic activity per 10 HPFs ( $\bar{\text{x}}\pm \text{s}$ )	11.0 ± 13.4	17.0 ± 16.0	0.273
Lymphovascular invasion			0.622
Present	4/31 (13%)	2/10 (20%)	
Absent	27/31 (87%)	8/10 (80%)	
Necrosis			0.621
Present	13/34 (38%)	6/13 (46%)	
Absent	21/34 (62%)	7/13 (54%)	
Recurrence			0.083
Yes	10/36 (28%)	7/12 (58%)	
No	26/36 (72%)	5/12 (42%)	
Disease-specific mortality			0.028^*^
Yes	2/36 (6%)	4/12 (33%)	
No	34/36 (94%)	8/12 (67%)	

^#^Only one case that harbored *NTRK2* fusion have been reported as far, so it has not been included in statistical analysis.

^*^p-value<0.05; Measurements were expressed as mean ± standard deviation ( $\bar{\text{x}}\pm \text{s}$ ). The clinicopathological features with statistically significant differences between the two groups have been highlighted.

## Discussion

In this study, we presented a rare case of NTRK-rearranged spindle cell neoplasm of the uterine cervix and performed a literature review encompassing 62 cases of this tumor entity in the female genital tract. Based on published reports, we summarized the clinicopathological characteristics, molecular alterations, and clinical outcomes, including our own case (1, 2, 6–8, 12–26). We believe that the significance of reporting this case is to expand the database of such tumors in the female reproductive tract, as the number of reported cases is small and the data available for analysis of pathological diagnosis, treatment, and prognosis are very limited.

Given the rarity of this tumor and the limited data currently available, documenting such cases is essential to expand the collective knowledge base regarding diagnosis, therapeutic implications, and prognostic evaluation in the female reproductive system.

### Pathogenesis

The three members of the neurotrophic tyrosine receptor kinase (*NTRK*) family (*NTRK1*, *NTRK2*, and *NTRK3*) encode TRKA, TRKB, and TRKC proteins, respectively (12). These receptors are physiologically expressed in the peripheral and central nervous systems and play critical roles in neural development and function (13–15). Upon ligand binding, the extracellular domains of NTRK receptors undergo dimerization, leading to autophosphorylation and activation of downstream signaling cascades, including the MAPK, PI3K, and PKC pathways. These pathways regulate essential cellular processes such as proliferation, differentiation, and survival (1, 27). *NTRK* gene rearrangements in tumors can result from either intrachromosomal or interchromosomal translocations. These rearrangements typically involve the fusion of the 5’ end of an *NTRK* gene with the 3’ end of a partner (chaperone) gene, producing a chimeric fusion protein. When the chaperone gene encodes a domain that facilitates dimerization, the resulting fusion protein can undergo constitutive dimerization without ligand binding. This ligand-independent activation leads to persistent stimulation of the downstream signaling pathways mediated by the TRK kinase domain, thereby promoting oncogenic transformation. This mechanism represents a key driver of tumorigenesis in NTRK-altered malignancies. Frequently reported fusion partners include LMNA, TPM3, PAN3, and ETV6 (14, 15). TRK receptor activation via NTRK fusion is recognized as a pan-cancer oncogenic mechanism, with an estimated incidence of 0.68% to 1% in adult soft tissue sarcomas (1, 8, 14, 27). NTRK rearrangements have been documented in a variety of soft tissue tumors in both adults and children, including infantile fibrosarcoma and congenital mesoblastic nephroma. However, such genetic alterations are exceedingly rare in gynecologic tumors.

### Pathological diagnosis

To date, few distinct morphological features have been validated as reliable diagnostic indicators for NTRK-rearranged spindle cell neoplasms. Therefore, definitive diagnosis largely depends on IHC analysis and molecular testing.

Among the IHC markers, TRK, CD34 and S-100(especially TRK) are considered valuable for raising suspicion of this tumor type and guiding further molecular investigations. Tumor cells typically exhibit diffuse cytoplasmic positivity for TRK. An exception to this pattern has been reported in a TRK-negative case harboring a *SPECC1L::NTRK3* fusion gene, which also demonstrated a loss of SMARCB1 (INI1) expression (23). In addition to the classic cytoplasmic staining, alternative TRK expression patterns have been described. For example, tumors with *LMNA::NTRK1* fusions may show nuclear membrane localization, while tumors with *TPM3::NTRK1* or *ETV6::NTRK3* fusions can exhibit membranous staining patterns (1, 14, 28). Although TRK immunostaining is a useful screening tool, it is important to note that TRK positivity does not always indicate *NTRK* gene rearrangements. Cases with TRK expression must be confirmed by molecular assays. For instance, some high-grade endometrial stromal sarcomas (ESS) have shown TRK positivity despite lacking any *NTRK* fusions (22). Therefore, confirmatory testing such as FISH, reverse transcription PCR (RT-PCR), or next-generation sequencing (NGS) is essential to establish the diagnosis. Additionally, the tumor frequently expresses S-100 and CD34 (29). S-100 expression was reported in 43 of 49 cases (88%) and CD34 in 34 of 45 cases (76%) (**Supplementary Table 2**). Thus, we recommend the use of TRK, CD34, and S-100 immunostaining as an accessible and cost-effective initial screening strategy for all uterine spindle cell tumors, particularly those lacking definitive endometrial stroma or smooth muscle differentiation, to facilitate the identification of NTRK-rearranged neoplasms.

### Differential diagnosis

As previously discussed, Many mesenchymal tumors of the uterus present with similar spindle cell morphology, making differential diagnosis based solely on conventional histopathology challenging. In this context, immunohistochemistry is an essential and practical tool to support accurate pathological classification. In our case, a broad range of differential diagnoses need to be considered based on the histological features, including low-grade and high-grade ESS, leiomyosarcoma, and IMT, among others. Low-grade ESS typically expresses CD10 with estrogen and progesterone receptors, whereas high-grade ESS may show immunoreactivity for cyclin D1 and/or BCOR. In contrast, our case was negative for myogenic markers such as desmin, caldesmon, calponin, and MyoD1, as well as for the IMT-associated marker ALK, supporting the exclusion of leiomyosarcoma, rhabdomyosarcoma and IMT. Adenosarcoma with sarcomatous overgrowth should also be excluded, as residual benign endocervical glands were observed among the tumor components in our case, but the characteristic periglandular sarcomatous cuffing typical of adenosarcoma was absent. For rare uterine malignant peripheral nerve sheath tumors (MPNSTs), which may show focal positivity for S-100 and CD34, the key distinction lies in their usual expression of neural markers. Some tumors previously diagnosed as MPNSTs have been reclassified as NTRK-rearranged spindle cell tumors by molecular testing (22).

### Prognosis

A recent study by Costigan (7) suggests that several indicators, including both morphological and genetic features, may predict poor prognosis, such as lymphovascular invasion, necrosis, mitotic counts ≥8 per 10 HPFs, and *NTRK3* fusion. Interestingly, tumors with *NTRK3* fusions were generally larger and exhibited higher mitotic activity compared to those with *NTRK1* fusions. They also tended to present at more advanced stages and had a higher likelihood of recurrence, suggesting a poorer outcome for these patients. Tumors exhibiting one or more of these features should be considered high-risk, whereas those lacking all of them may be classified as low-risk (7). According to these criteria, our case should be regarded as high-risk due to the presence of necrosis and active mitosis, and warrants prolonged follow-up despite no evidence of recurrence to date. In the female genital tract, 75% of reported cases harbored *NTRK1* fusions, with fusion partners including *TPM3*, *TPR*, *IRF2BP2*, *C16ORF72*, *LMNA*, *NUMA1*, and *TRIM67*. Notably, all patients with *TPM3::NTRK1* fusions (accounting for 59% of cases) were alive at the time of follow-up. These findings suggest that fusion type may correlate with survival outcomes, although further investigation is warranted.

Furthermore, it has been proposed that some NTRK-rearranged sarcomas with high-grade nuclear features may harbor *TP53* mutations (7). In general, *TP53* mutations result in a mutant-type immunostaining pattern, which may serve as a surrogate marker for underlying genetic alteration. *TP53* mutations have been associated with high-grade nuclear features and poor prognosis in other tumors, such as endometrial carcinoma. However, the relationship between p53 expression patterns and prognosis remains unclear in NTRK-rearranged sarcomas of the female genital tract, highlighting the need for further studies exploring the prognostic significance of different p53 staining patterns.

### Treatment

Most patients with NTRK-rearranged spindle cell neoplasms underwent surgical resection followed by adjuvant chemotherapy, with or without radiotherapy. As these tumors are typically negative for hormone receptors and rarely involve the ovaries, ovarian preservation does not appear to adversely affect prognosis (7, 12). The necessity of oophorectomy in young patients with NTRK-rearranged sarcomas remains uncertain. Studies have demonstrated that TRK inhibitors, such as entrectinib and larotrectinib, are highly effective, particularly in patients with advanced or metastatic disease. According to reports in the literature, 109 patients with *NTRK* gene fusions received larotrectinib treatment, achieving an investigator-assessed overall response rate of 81% (95% CI: 72%–88%). Among them, 63% experienced partial responses, while 17% achieved complete responses. Based on these results, the U.S. Food and Drug Administration (FDA) approved larotrectinib in 2018 for the treatment of solid tumors harboring *NTRK* gene fusions (4, 6, 10, 16, 17, 30). In our case, the patient underwent radical hysterectomy followed by adjuvant chemotherapy and radiotherapy, but has not received targeted therapy, as the disease was diagnosed at an early stage and no recurrence has been observed to date. Therefore, we are currently unable to assess the efficacy of TRK inhibitors in this case. It is worth noting that follow-up data for this tumor type remain limited, highlighting the need for larger, long-term studies to further evaluate treatment strategies.

## Conclusion

In summary, primary NTRK-rearranged spindle cell neoplasm in the female genital tract is rare. Diagnosis based solely on clinical presentation or routine histopathology is challenging. Accurate pathological identification requires immunohistochemistry and molecular testing. Although the number of reported cases remains limited, affected patients may benefit from treatment with TRK inhibitors. Therefore, precise diagnosis is crucial for both gynecologists and pathologists in optimizing patient management.

## Data Availability

The original contributions presented in the study are included in the article/**Supplementary Material**. Further inquiries can be directed to the corresponding author.
