# Supplementary material for: NTRK-rearranged spindle cell neoplasm of the female genital tract: case report and literature review
Source: Front Oncol. 2025 Aug 27;15:1525722. doi: 10.3389/fonc.2025.1525722 (PMC12420217; doi:10.3389/fonc.2025.1525722)
Supplement: Supplementary file 2 [file Table1.docx]

**Supplementary Table 1. Information of primary antibodies**

| Antibody | Clone | Manufacturer | Dilution | Platform |
| --- | --- | --- | --- | --- |
| TRK | 32514C2 | Abcarta(Suzhou, China) | Ready-to-Use | Leica Bond III |
| BCOR | C-10 | MXB Biotech(Fuzhou,China) | Ready-to-Use | Leica Bond III |
| Desmin | MX046 | MXB Biotech(Fuzhou,China) | 1:400 | ZSGB-BIO UltraPATH |
| Caldesmon | h-CALD | MXB Biotech(Fuzhou,China) | 1:150 | ZSGB-BIO UltraPATH |
| Calponin | MX023 | MXB Biotech(Fuzhou,China) | 1:3000 | ZSGB-BIO UltraPATH |
| SMA | 1A4 | MXB Biotech(Fuzhou,China) | 1:100 | ZSGB-BIO UltraPATH |
| MyoD1 | MX049 | MXB Biotech(Fuzhou,China) | Ready-to-Use | Leica Bond III |
| Myogenin | Rabbit polyclonal | MXB Biotech(Fuzhou,China) | Ready-to-Use | Leica Bond III |
| CD10 | MX002 | MXB Biotech(Fuzhou,China) | 1:100 | Leica Bond III |
| Cyclin D1 | SP4 | MXB Biotech(Fuzhou,China) | Ready-to-Use | ZSGB-BIO UltraPATH |
| P53 | MX008 | MXB Biotech(Fuzhou,China) | 1:500 | Leica Bond III |
| ER | SP1 | Ventana(Tucson, USA) | Ready-to-Use | VentanaBenchmark Ultra |
| PR | 1E2 | Ventana(Tucson, USA) | Ready-to-Use | VentanaBenchmark Ultra |
| ALK | 5A4 | MXB Biotech(Fuzhou,China) | 1:200 | ZSGB-BIO UltraPATH |
| S-100 | 4C4.9 | MXB Biotech(Fuzhou,China) | 1:200 | Leica Bond III |
| CD34 | QBEnd/10 | MXB Biotech(Fuzhou,China) | 1:100 | ZSGB-BIO UltraPATH |
| Ki67 | MXR002 | MXB Biotech(Fuzhou,China) | 1:800 | Leica Bond III |
