# Supplementary material for: NTRK-rearranged spindle cell neoplasm of the female genital tract: case report and literature review
Source: Front Oncol. 2025 Aug 27;15:1525722. doi: 10.3389/fonc.2025.1525722 (PMC12420217; doi:10.3389/fonc.2025.1525722)
Supplement: Supplementary file 3 [file Table2.docx]

| **Supplementary Table 2.** Previously reported cases of *NTRK*-rearranged uterine sarcomas. | | | | | | | | | | | | |
| --- | --- | --- | --- | --- | --- | --- | --- | --- | --- | --- | --- | --- |
| Case | Series | Age (years) | Site | Stage | Recurrence | Outcome | Size (cm) | Atypia | Necrosis | LVI | Mitoses/10 HPF | Fusion |
| 1 | Chiang^1^ | 46 | cervix | ⅠB | Y | AWD | 9.3 | 2 | Y | N | 15 | *TPM3::*  *NTRK1* |
| 2 | Goulding^13^ | 13 | cervix | ⅠB | N | NED | 9.2 | 1 | NK | NK | NK | *TPM3::*  *NTRK1* |
| 3 | Boyle^15^ | 42 | cervix | ⅠB | N | NED | 5.2 | 1 | N | NK | 8 | *TPM3::*  *NTRK1* |
| 4 | Croce^4^ | 39 | cervix | NK | N | LTFU | NK | 1 | Y | N | 3 | *TPM3::*  *NTRK1* |
| 5 | Croce^4^ | 44 | cervix | ⅠA | N | NED | 4.5 | 2 | N | N | 3 | *TPM3::*  *NTRK1* |
| 6 | Croce^4^ | 23 | cervix | ⅠA | N | NED | 3 | 2 | Y | N | 5 | *TPM3::*  *NTRK1* |
| 7 | Rabban^11^ | 30 | cervix | ⅠA | Y | AWD | 2.5 | 1 | N | N | 18 | *TPM3::*  *NTRK1* |
| 8 | Wells^25^ | 30 | corpus | ⅠB | NK | NED | 2.5 | 1~2 | N | N | 2 | *TPM3::*  *NTRK1* |
| 9 | Gatalica^2^ | NK | cervix | NK | NK | NK | NK | NK | NK | NK | NK | *TPM3::*  *NTRK1* |
| 10 | Devereaux^17^ | 39 | cervix | ⅠB | N | NED | 5.8 | 1~2 | Y | N | 12 | *TPM3::*  *NTRK1* |
| 11 | Devereaux^17^ | 66 | cervix | ⅠA | N | NED | 1.5 | 1~2 | N | N | 1 | *TPM3::*  *NTRK1* |
| 12 | Nilforoushan^6^ | 52 | cervix | ⅠA | N | NED | 1.3 | 1 | NK | NK | 1 | *TPM3::*  *NTRK1* |
| 13 | Tsai^24^ | 47 | cervix | NK | Y | AWD | 2.7 | 2~3 | Y | NK | 3 | *TPM3::*  *NTRK1* |
| 14 | Tsai^24^ | 53 | cervix | NK | Y | AWD | 6.8 | 2~3 | Y | NK | 26 | *TPM3::*  *NTRK1* |
| 15 | Costigan^7^ | 35 | cervix | ⅠA | NK | LTFU | 3.5 | 2 | N | N | 5 | *TPM3::*  *NTRK1* |
| 16 | Costigan^7^ | 39 | cervix | NK | NK | LTFU | NK | 2~3 | Y | N | 16 | *TPM3::*  *NTRK1* |
| 17 | Costigan^7^ | 24 | cervix | ⅠA | N | NED | NK | 2~3 | N | N | 2 | *TPM3::*  *NTRK1* |
| 18 | Costigan^7^ | 26 | cervix | NK | NK | LTFU | 8 | 2 | N | N | 1 | *TPM3::*  *NTRK1* |
| 19 | Grant^18^ | 42 | cervix | ⅠA | Y | AWD | 5.2 | 1 | N | N | 4 | *TPM3::*  *NTRK1* |
| 21 | Croce^4^ | 30 | cervix | ⅠA | N | NED | 2.5 | 1 | N | N | 50 | *TPM3::*  *NTRK1* |
| 22 | Croce^4^ | 33 | cervix | ⅠA | N | NED | 5 | 1 | N | Y | 1 | *TPM3::*  *NTRK1* |
| 23 | Croce^4^ | 23 | cervix | ⅡA | Y | NED | 2.8 | 3 | Y | N | 32 | *TPM3::*  *NTRK1* |
| 24 | ^*^Huang^19^ | 16~82  (47) | cervix | NK | N | AWD | 4~12  (6.57) | 2 | NK | NK | 2~15  (5) | *TPM3::*  *NTRK1* |
| 25 | ^*^Huang^19^ | 16~82  (47) | cervix | NK | Y | AWD | 4~12 (6.57) | 1 | NK | NK | 2~15  (5) | *TPR::NTRK1* |
| 26 | ^*^Huang^19^ | 16~82  (47) | cervix | NK | N | AWD | 4~12  (6.57) | 2 | NK | NK | 2~15  (5) | *TPR::NTRK1* |
| 27 | Chiang^1^ | 42 | cervix | ⅠB | Y | NED | 2.6 | 3 | Y | N | 30 | *TPR::NTRK1* |
| 28 | Rabban^11^ | 49 | cervix | NK | N | NED | 1.8 | 1~2 | N | N | 0 | *TPR::NTRK1* |
| 29 | Devereaux^17^ | 40 | cervix | ⅠA | N | NED | 2 | 2 | N | N | 1 | *TPR::NTRK1* |
| 30 | Costigan^7^ | 47 | cervix | ⅠB | NK | DSD | 7.8 | 1 | N | Y | 8 | *TPR::NTRK1* |
| 31 | Costigan^7^ | 30 | cervix | ⅡB | N | NED | 4.0 | 1~2 | N | N | 4 | *TPR::NTRK1* |
| 32 | Costigan^7^ | 16 | cervix | ⅠA | N | NED | NK | 2 | N | N | 6 | *TPR::NTRK1* |
| 33 | Costigan^7^ | 42 | cervix | ⅠB | N | NED | 5.6 | 3 | N | N | 26 | *TPR::NTRK1* |
| 34 | Grant^18^ | 32 | cervix | ⅠB | Y | DSD | 8 | 1 | N | N | 48 | *TPR::NTRK1* |
| 35 | Devereaux^17^ | 35 | corpus | ⅠB | N | NED | 9.4 | 2~3 | N | N | 5 | *C16orf72::*  *NTRK1* |
| 36 | Costigan^9^ | 35 | corpus | ⅠB | NK | LTFU | 5.1 | 1~2 | N | N | 1 | *C16orf72::*  *NTRK1* |
| 37 | Devereaux^17^ | 37 | cervix | ≥ⅠB | N | LTFU | 6.3 | 1~2 | Y | N | 2 | *IRF2BP2::*  *NTRK1* |
| 38 | Costigan^7^ | 46 | cervix | ⅠB | NK | LTFU | 10 | 3 | Y | Y | 3 | *IRF2BP2::*  *NTRK1* |
| 39 | Chiang^1^ | 27 | corpus | ⅠB | N | NED | 16.3 | 2 | N | N | 7 | *LMNK::*  *NTRK1* |
| 40 | Szalai^23^ | 43 | cervix | NK | Y | AWD | 8 | 1 | Y | Y | 26 | *NUMA1::*  *NTRK1* |
| 41 | ^*^Huang^19^ | 16~82  (47) | cervix | NK | N | AWD | 4~12  (6.57) | 2 | NK | NK | 2~15  (5) | *TRIM67::*  *NTRK1* |
| 42 | ^*^Huang^19^ | 16~82  (47) | cervix | NK | N | AWD | 4~12  (6.57) | 2 | NK | NK | 2~15  (5) | *NTRK1* |
| 43 | ^*^Huang^19^ | 16~82  (47) | cervix | NK | N | AWD | 4~12  (6.57) | 3 | NK | NK | 2~15  (5) | *NTRK1* |
| 44 | ^*^Huang^19^ | 16~82  (47) | cervix | NK | N | AWD | 4~12  (6.57) | 2~3 | NK | NK | 2~15  (5) | *NTRK1* |
| 45 | Moh, Costigan^7^ | 69 | corpus | ⅠB | N | NED | 7 | 3 | Y | Y | 15 | *WWOX::*  *NTRK2* |
| 46 | Dang^12^ | 33 | cervix | ⅡA2 | Y | DSD | 4.5 | 1~2 | N | N | ＞30 | *EML4::*  *NTRK3* |
| 47 | Croce^4^ | 26 | cervix | ⅠB | N | AWD | 12 | 2 | Y | N | 3 | *EML4::*  *NTRK3* |
| 48 | Costigan^7^ | 26 | cervix | ⅡB | Y | NED | 12 | 1 | N | N | 4 | *EML4::*  *NTRK3* |
| 49 | Chiang^1^ | 47 | cervix | ⅠB | Y | DSD | 14 | 2 | Y | N | 12 | *RBPMS::*  *NTRK3* |
| 50 | Grant^18^ | 34 | cervix | ⅣB | Y | AWD | 14.6 | 3 | Y | Y | 40 | *SPECC1L::*  *NTRK3* |
| 51 | Razack^22^ | 28 | cervix | ⅠB | N | DSD | 11.3 | ＜3 | Y | N | 14 | *SPECC1L::*  *NTRK3* |
| 52 | Rabban^8^ | 24 | cervix | ⅠB | Y | AWD | 15 | 1~2 | N | Y | 12 | *SPECC1L::*  *NTRK3* |
| 53 | Hodgson^5^ | 55 | cervix | ⅠA | N | NED | 1.6 | 1~2 | N | N | 0 | *SPECC1L::*  *NTRK3* |
| 54 | Gatalica^2^ | NK | uterus | NK | NK | NK | NK | NK | NK | NK | NK | *SPECC1L::*  *NTRK3* |
| 55 | Nilforoushan^6^ | 54 | cervix | ⅠB | Y | AWD | 5.4 | 2 | Y | NK | 40 | *SPECC1L::*  *NTRK3* |
| 56 | Costigan^7^ | 61 | cervix | ⅠB | N | NED | 7 | 2 | Y | N | 4 | *SPECC1L::*  *NTRK3* |
| 57 | Michal^20^ | 26 | uterus | ≥ⅠB | N | NED | 23 | 1 | N | NK | 0 | *STRN::*  *NTRK3* |
| 58 | Costigan^7^ | 26 | cervix | ≥ⅠB | Y | DSD | 5.5 | 1 | N | N | 43 | *TFG::*  *NTRK3* |
| 59 | Wong^12^ | 31 | cervix | NK | NK | LTFU | 9 | NK | N | NK | 15 | *NTRK3* |
| 60 | Costigan^7^ | 26 | cervix | NK | Y | DSD | NK | 1~2 | N | N | 23 | NK |
| 61 | Costigan^7^ | 42 | cervix | NK | NK | LTFU | NK | 1~2 | N | N | 7 | NK |
| ^*^The literature lacks descriptions of the clinical characteristics of individual cases. | | | | | | | | | | | | |
| LTFU = lost to follow up; AWD indicates alive with disease; NED = no evidence of disease; DSD = disease-specific death; NK = not known; HPF=high power fields; LVI = lymphovascular invasion | | | | | | | | | | | | |
